# Significance of rs1271572 in the estrogen receptor beta gene promoter and its correlation with breast cancer in a southwestern Chinese population

**DOI:** 10.1186/1423-0127-20-32

**Published:** 2013-05-28

**Authors:** Li Chen, Yan Liang, Juhui Qiu, Lingling Zhang, Xianchun Chen, Xiangdong Luo, Jun Jiang

**Affiliations:** 1 Breast Disease Center, Southwest Hospital, Third Military Medical University, Chongqing, 400038, P.R. China; 2 Burn Research Institute, Southwest Hospital, Third Military Medical University, Chongqing, 400038, P. R. China

**Keywords:** Breast cancer, SNP, Estrogen receptor beta, Prognosis, Chinese

## Abstract

**Background:**

To characterize single nucleotide polymorphisms (SNPs) within the promoter region of the estrogen receptor beta (ERβ) gene and to analyze the association of ERβ SNPs with susceptibility to breast cancer. Genotype frequencies of five SNPs (rs3020449, rs3020450, rs2987983, rs1271572 and rs1887994) in the promoter region of the ERβ gene in 873 women with breast cancer, 645 women with fibroadenoma and 700 healthy women were determined using an allele-specific tetra-primer polymerase chain reaction (PCR). Kaplan-Meier survival analysis was performed to evaluate the association of selected rs1271572 with prognosis in breast cancer. Electrophoretic mobility-shift assays were conducted to explore the binding of SNP rs1271572 containing probes to transcriptional factor Ying Yang 1 (YY1).

**Results:**

Women with the homozygous TT genotype of rs1271572 had a significantly higher risk in developing breast cancer. Breast cancer patients with the TT genotype of rs1271572 had lower five-year survival rates than those with other genotypes and were more likely to suffer brain metastases. The rs1271572 G→T SNP abrogated YY1 binding and reduced the transcription activity of the promoter 0 N in the ERβ gene *in vitro*.

**Conclusions:**

TT genotype of rs1271572 is associated with increased risk for breast cancer in Chinese women and is associated with unfavored prognosis in Chinese breast cancer patients. TT genotype of rs1271572 inhibited expression of ERβ gene by down regulating transcriptional activity of the promoter 0 N in the ERβ gene. Our data revealed that the TT genotype of rs1271572 resulted in loss of the YY1 binding site and reduced the transcription activity of the promoter 0 N in the ERβ gene.

## Background

Breast cancer is a worldwide health problem among women and causes an estimated 450,000 deaths annually [1]. In China,breast cancer is the most prevalent cancer in women and ranks as the 6th leading cause of death in Chinese women [2,3]. Single nucleotide polymorphisms (SNPs) are the most frequent sequence variations in the human genome [4]. Evidence from population studies have shown that some SNPs can affect breast cancer risk and survival.

ERβ (estrogen receptor β) plays a significant role in suppressing breast cancer cell proliferation [5] and acts as a negative modulator of ERα activity [6]. ERβ expression declines during breast tumorigenesis [7]; however, the mechanisms of ERβ down-regulation in breast cancer remain unclear. A few studies have reported an association between genotypic polymorphisms of ERβ with breast cancer susceptibility [8-10]. However, most of the population studies were performed in Europeans and Americans and the results have been inconsistent [11,12]. Currently, no such association studies have been performed in the Chinese populations.

SNPs located in exons may alter protein function, whereas SNPs in the gene promoter can modify gene expression levels [13,14]. Hirata et al. reported that transcription of the human ERβ gene occurs from at least two different promoters, named promoter 0 N and promoter 0 K [15]. Further studies have shown that transcripts from promoter 0 N are more prominent than those from promoter 0 K in normal breast epithelial cells and in a panel of breast cancer cell lines [16]. Based on a bioinformatics analyses of all the polymorphisms identified in the putative promoter of the ERβ gene, we selected five SNPs near the transcription start site of promoters 0 N of the ERβ gene (rs3020449 C/T, rs3020450 A/G, rs2987983C/T, rs1271572 G/T and rs1887994 G/T) as targets for the present study and characterized the five SNPs in the putative promoter of the ERβ gene in a population of Chinese women from southwest China. We also investigated whether an association exists between breast cancer risk and SNP rs1271572 G/T in the ERβ gene promoter and determined the effect of this SNP on ERβ gene expression.

## Methods

### Ethics statement

The study was approved by the Ethical Committee of the Third Military Medical University and the Hospital Clinic Ethics Review Committee. Written informed consent was obtained from all participants involved in the study.

### Study subjects

From January 2002 to December 2004, all the sporadic breast cancer patients with invasive ductal carcinoma who were selected to undergo surgery and adjuvant chemotherapy (cyclophosphamide 100 mg/m^2^ D1, doxorubicin 30 mg/m^2^ D1,8, and fluorouracil 500 mg/m^2^ D1,8; all drugs given intravenously (IV) every 28 days, for a total of six cycles) were eligible for the present study. Diagnosis was confirmed using histologic examination of all specimens. Exclusion criteria included pregnancy, non-surgical candidates, patients unwilling to undergo treatment, patients with a family history of breast cancer, patients with congestive heart failure, ischemic heart disease, other malignancies, severe hepatic or renal dysfunction, altered mental status or patients who were over 70 years old. The patients (n = 873; median age 45.8 ± 12.5 years, range 22–70 years) were diagnosed at our Breast Disease Center at the Third Military Medical University and at the Affiliated Hospital of North Sichuan Medical College, China. Eighteen of the 873 patients were excluded because tetra-primer ARMS PCR failed. Since it was difficult to obtain a sufficient number of normal breast tissue samples, we chose to use tissues from fibroadenoma patients as controls in a scoring system for ERβ expression. The patients with fibroadenomas (n = 645; median age 44.5 ± 10.6 years, range 18–69 years) were enrolled after receiving appropriate informed consent. Fibroadenoma is not a premalignant disease. 700 healthy female blood donors (median age 41.5 ± 12.1 years, range 20–59 years) with no history of any type of cancer or any relatives with a history of breast cancer were recruited for the present study as the second control group. The average age of incidence of breast cancer is 45.8. This is one of the criteria for selecting 700 cases of healthy controls. According to regulations in China, people over 60 years of age are not allowed to donate blood. Other factors in control groups are matched with those in breast cancer groups. In addition, the control subjects (patients with fibroadenomas and healthy female blood donors) should be drawn from the same population.

### Bioinformatics analyses of the ERβ gene promoter

Our hypothesis is that SNPs in the promoter of the ERbeta gene could affect its expression, which could be a risk factor for breast cancer. Thus, we searched SNPs near the promoter of ER beta. The transcription of the human ERβ gene occurs from at least two different promoters, promoter 0 N and promoter 0 K [15]. AS transcripts from promoter 0 N were found to be more prominent than those from promoter 0 K [15,16], we referred to the NCBI database of known SNPs (http://www.ncbi.nlm.nih.gov/snp/?term=ESR2, NCBI36/hg18) and selected five SNPs from the putative promoter region near the transcription start site of promoters (as shown in Additional file 1: Figure S1) 0 N: rs3020449, rs3020450, rs2987983, rs1271572 and rs1887994 that were also studied by others [8,17-20].

### Tetra-primer ARMS PCR

Genomic DNA from blood was isolated using a Wizard Genomic DNA Purification Kit (Promega, USA) according to the manufacturer’s instructions. The genomic DNA was resuspended in 50 μL PCR buffer (GoTaq buffer, Promega, USA), containing 0.5% Tween 20, 10 mAnson units proteinase K (Merck, Germany) and incubated at 50°C overnight. The mixture was then heated for 10 min at 95°C to inactivate the enzyme.

Allelic-specific tetra-primer amplification was performed on the genomic DNA using a tetra-primer ARMS PCR approach. Each PCR reaction was carried out in a total volume of 10 μl, containing 30 ng of template DNA, 10 pmol of each inner primer, 1 pmol of each outer primer, 200 μM dNTP, and 0.5 units Go Taq polymerase (Promega, USA). The reaction was overlaid with 5 μl of liquid paraffin. The PCR cycling conditions for the detection of rs3020449 and rs3020450 were as follows: 95°C for 5 min, then 33 cycles of 95°C for 1 min, 61°C for 1 min, and 72°C 1 min, followed by 72°C for 5 min. For detecting rs2987983, rs1271572 and rs1887994, the following cycling conditions were utilized: 94°C for 5 min, 33 cycles of 94°C for 30 s, 59°C for 1 min and 72°C for 1 min, followed by 72°C for 5 min. PCR products were mixed with 2 μl of loading buffer and analyzed using 3% agarose gel electrophoresis. The primers used for the PCR reaction are listed in Additional file 2: Table S1. Allelic-specific PCR product sizes were 353/250 bp (C/T) for SNP rs3020449, 209/ 419 bp (A/G) for rs3020450, 276/164 bp (C/T) for rs2987983, 276/133 bp (G/T) for rs1271572 and 229/418 bp (G/T) for SNP rs1887994. The genotyping success rate was 97.8%. As a quality control for genotyping, in each PCR reaction two previously characterized DNA samples representing the heterozygous and the two homozygous genotypes were analyzed in addition to the unknown samples. Genotyping was performed blinded without knowledge of the patients’ clinical data.

### Cell culture

Human breast cancer cell lines MDA-MB-231, MCF-7, MDA-MB-468, BT-549 and ZR75-30 (purchased from the American Type Culture Collection, Manassas, VA, USA), were maintained in modified MEM media supplemented with 10% fetal bovine serum (FBS), 2 mM L-glutamine and 20 lg/mL gentamycin. Cell cultures were grown at 37°C in a humidified atmosphere with 5% CO_2_ in a Hereaus CO_2_ incubator. To culture primary breast cancer cells, clinical resected breast cancer specimens were sheared to fractions, digested with trypsin/EDTA (0.05%/0.02% in PBS) for 20–30 min at 37°C, followed by three washes with culture medium containing 10% FBS. Following filtration through 56 μm gauze, the single-cell suspension was then counted and seeded in six-well plates. After one week, the cancer cell clones were selected for culture. The primary breast cancer cells were identified using pathological examination.

### Plasmid construction, YY1 knock down, transient transfection and luciferase reporter assays

The cDNA of promoter 0 N plus the partial upstream sequence of the intron up to the ATG transcriptional starting site in exon 1 (ESR2-0 N + −2316 ~ +1) and the cDNA of promoter 0 K plus the partial upstream sequence of the intron up to the ATG of exon 1 (ESR2-0 K + −1868 ~ +1) (as shown in Additional file 3: Figure S2) were amplified from genomic DNA and the corresponding fragments were then subcloned into the Sac I and Hind III sites of the luciferase reporter vector, pGL3-basic (Promega) [10], to create pESR2-0 N-G-Luc (containing the G allele of rs1271572), pESR2-0 N-T-Luc (containing the T allele of rs1271572) and pESR2-0 K-Luc vectors. All vectors were confirmed by sequencing. To specifically knock down YY1, YY1-specific siRNA oligo sequences were used as follows: the siRNA target sequence: GACGACGACTACATTGAACAA; the siRNA sense: oligo r (CGACGACUACAUUGAACAA)dTdT; and the siRNA antisense oligo: r(UUGUUCAAUGUAGUCGUCG) dTdC.

Transient transfection was performed using FuGENE 6 reagent (Roche Applied Science) as described previously [5]. In brief, breast cancer cells growing from indicated cell lines or primary cultures were seeded into a 24-well plate at a density of 1 × 10^5^ cells/well or in 6-well plate at a density of 1× 10^6^ cells/well and were allowed to grow for 24 hours. 1.0 μg of reporter gene constructs pRL alone or in combination with 5 nM YY1-specific siRNA oligos were transiently transfected into these cells. 48 hours post transfection, cells were rinsed with PBS and then lysed in 1× passive lysis buffer. Luciferase activity was determined using a dual-luciferase reporter assay kit (Promega). The ratio of firefly luciferase activity was normalized to Renilla luciferase activity. The pGL3-control (Promega) was also used as an additional control in these experiments.

### Electrophoretic mobility-shift assay (EMSA)

Nuclear extract preparation and EMSAs were performed as previously described [21] with slight modifications. In brief, a thawed breast cancer cell pellet was first lysed in ice-cold Buffer A (25 mm Tris–HCl, pH 7.5, 50 mm KCl, 2 mm MgCl_2_, 5 mm dithiothreitol, and inhibitors of phosphatases). After removing supernatants by centrifugation, buffer C (20 mm Tris–HCl, pH 7.5, 0.42 m NaCl, 1.5 mm MgCl_2_, 25% sucrose, 1 mm dithiothreitol, and inhibitors of phosphatase) was added and gently shaked for 30 min at 0°C. The supernatants were collected and concentration of nuclear proteins was measured. The oligonucleotides probes containing the two SNP alleles of the human ERβ gene promoter region were as follows: probe–rs1271572G (with the G allele), 5’-TGTGACACTGGGGGGGTCTCACAATGGCCT-3’; and probe–rs1271572T (with the T allele), 5’-TGTGACACTGGGGGGTTCTCACAATGGCCT-3’. The double-stranded DNA probes were end-labeled with γ- ^32^P ATP and T4-polynucleotide kinase (Promega). The nuclear protein-DNA binding reaction was performed at 37°C for 20 min in a total volume of 20 μl containing 10 μg nuclear extract proteins and 0.2 ng of labeled double probes or a 50-fold non-labeled competitor probe. The DNA-protein complexes were resolved by electrophoresis through a 4% poly-acrylamide gel. Gels were dried and the labeled complexes were detected by autoradiography.

### Immunohistochemistry

The breast specimens from patients with breast cancer or fibroadenoma were fixed in a 0.1 M phosphate-buffered 10% formaldehyde solution for 24 h, dehydrated, and embedded in paraffin (5 μm sections were used). The primary antibody dilutions found to be optimal for this study were 1:500 for ERβ (mAb, Chemicon International). All data on ERα, PgR, and HER2 status were retrieved from pathology reports performed by a pathologist (positives are defined when more than 10% of tumor cells are stained). The specificity of the immunohistochemical procedures was checked using negative and positive control sections.

A scoring system for ERβ expression in the tissues was used as follows: no staining (0 points), weak staining (1 point, staining in less than 20% of tumor cells), low staining (2 points, staining in 21–40% of tumor cells), medium staining (3 points, staining in 41–60% of tumor cells), and high staining (4 points, staining in over 61% of tumor cells) tissues. Each tissue has three different slides. The typical slice including the center and edge of cancer were chosen and total positive cancer cell rate was calculated as the score for that tissue by a pathologist. A total of 1,483 immunohistochemically stained ERβ tissues (from 628 fibroadenoma and 855 breast cancer) underwent central review.

### Statistical analyses

The allelic and genotypic frequencies for each SNP were calculated, and deviation from the Hardy–Weinberg equilibrium was estimated using a *χ*^2^ test. The differences in allelic and genotypic frequencies between the breast cancer group, the fibroadenoma group, and the healthy blood donor group were estimated and statistical tests for association (95% confidence interval [CI]) and for significance were performed using SPSS (version 14) for Windows (SPSS, Inc., Chicago, IL) and SHEsis (http://analysis.bio-x.cn/myAnalysis.php). The results were considered to be significant when *p* < 0.05.

Cause-specific survival was defined as the time from diagnosis to death if the patient died of breast cancer, or to last known contact. Data for patients who died from causes other than breast cancer, who missed follow-up or for whom contact was interrupted, were censored. The Kaplan–Meier method with a log-rank test was used to establish the significance of genotypes of rs1271572 as predictors of Survival Probability (SP). For the 631 women with breast cancer, the same test was used to compare differences between the different groups defined by the G/T genotypes of rs1271572 (promoter of ERβ gene), by their ERβ–positive and ERβ–negative status, their ERα–positive and ERα–negative status, their HER2–positive and HER2–negative status, or their PgR–positive and PgR–negative status.

Cox proportional hazards models were used to test for association of survival times of patients to their rs1271572 genotypes and to clinical characteristics, such as menopause, age, tumor size, nodal status, breast cancer grade, and ERα/HER2/PgR status. The 95% CI was used to quantify the relationship between survival time and each independent factor. All tests were 2-sided, and *p* values <0.05 were considered to be statistically significant.

## Results and discussion

### The rs1271572 G → T genotype is associated with breast cancer

As a negative regulator of ERα, ERβ plays a critical role in breast cancer development. A number of common polymorphisms have been identified in the ERβ gene [11,12], with variable degrees of evidence of their direct biological significance and their association with human diseases [22-25]. A total of five SNPs in the promoter region of the ERβ (ESR2) gene were selected (http://www.ncbi.nlm.nih.gov/snp) as candidates for conferring variations in all 1,518 patients (873 with breast cancer and 645 with breast fibroadenoma) and in the 700 healthy blood donors. The tetra-primer ARMS-PCR method was successfully applied to five different SNPs in the promoter of the ERβ gene, i.e. the rs3020449 C → T, rs3020450 A → G, rs2987983 C → T, rs1271572 G → T and rs1887994 G → T polymorphisms. The genotypes determined using this method were consistent with those determined by the classical restriction endonuclease digestion method [26].

Genotyping results for the selected 5 SNPs were summarized in Additional file 4: Table S2. Specifically, the frequencies of the genotypes for rs3020449, rs3020450 and rs1271572 were in Hardy-Weiberg equilibrium (http://analysis.bio-x.cn/myAnalysis.php) (*p* > 0.05) in this set of study population. No significant differences were observed for the homozygous/heterozygous genotype frequencies of SNPs rs3020449 and rs3020450 among the three groups. A higher frequency of the homozygous TT genotype in rs1271572 was observed in women with breast cancer compared with those with fibroadenoma (*p* = 0.00019) or healthy controls (p = 0.0008). There was no significant difference in the TT genotype frequencies between the fibroadenoma group and the blood donors group. In addition, the genotype–phenotype association suggests that the TT genotype of rs1271572 is a risk factor for breast cancer development (cancer group *vs*. blood donors group: Odds Ratio (OR) = 0.546, [95%CI 0.404–0.739], χ^2^ = 15.54, *p* = 0.00008; and cancer group *vs*. fibroadenoma group: OR = 0.558, [95%CI 0.409–0.759], χ^2^ = 13.89, *p* = 0.00019).

The defect in PCR primer resulted in experimental failure of the rs2987983. Indeed, the relationship between SNPs in the promoter region and expression of ERbeta has seldom been reported in China. Our studies are mainly from a foreign population, which may cause rs1887994 selection failure. In addition, we did not do Bonferroni adjustment, as only a few sites were analyzed.

Our analyses of the allelic frequencies of the five selected ERβ SNPs in these study subjects revealed that women with breast cancer more frequently carried the T allele of SNP rs1271572 [cancer 45.03% *vs*. blood donors 38.35%, OR = 1.310, 95% CI (1.060–1.619), χ^2^ = 6.27, *p* = 0.01229; cancer 45.03% *vs*. fibroadenoma 38.38% OR = 1.331, 95% CI (1.072–1.654), χ^2^ = 6.69, *p* = 0.00969]. In contrast, no significant difference in the allelic frequencies of SNPs rs3020449 and rs3020450 were detected among the three groups.

The SNPs in the promoter 0 N region of the ERβ gene may affect breast cancer risk. Treeck provided evidence that the CC genotype of rs2987983 could be a risk factor for breast cancer development [8]. A recent report showed that homozygotes for the ERβ gene rs1271572/T are risk factors for postmenopausal breast cancer treated with hormone therapy [18]. The rs1271572 polymorphism is also associated with prostate cancer risk among Chinese men [20]. However, another study showed that none of the SNPs in the ERβ gene promoter (including rs1271572) were independently associated with breast cancer risk [19]. Thus, the association of rs1271572 with breast cancer remains controversial. But we reported that the TT genotype of rs1271572 was present at a significantly higher frequency in breast cancer patients than in fibroadenoma patients or in the blood donor control group (p < 0.001), suggesting for the first time that the TT genotype of rs1271572 in the ERβ gene promoter might be an important risk factor for breast cancer in Chinese women.

### TT genotype of rs1271572 was associated with downregulation of ERβ expression in breast cancer

**Figure 1:**
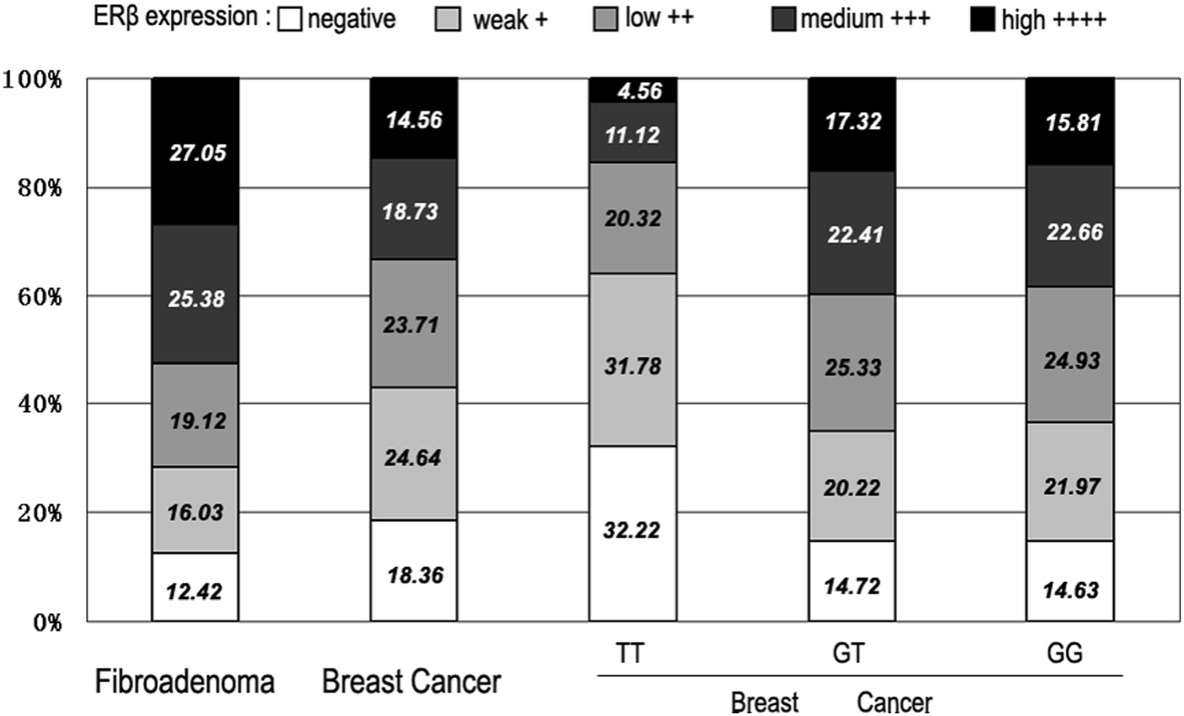
**Results of ER**β **staining in five consecutive trials.** Human breast cancer sections immunostained for ERβ were subdivided using a scoring system for ERβ expression into negative (no staining), weak (<20% staining), low (21–40% staining), medium (41–60% staining), and high (>61% staining) expression. The percentage of sections in each expression level is indicated. The first two columns showed the results for all the fibroadenoma (column 1) and breast cancer (column 2) sections. The last three columns showed the results for all breast cancer sections in each of the genotypes of rs1271572.

**Figure 2:**
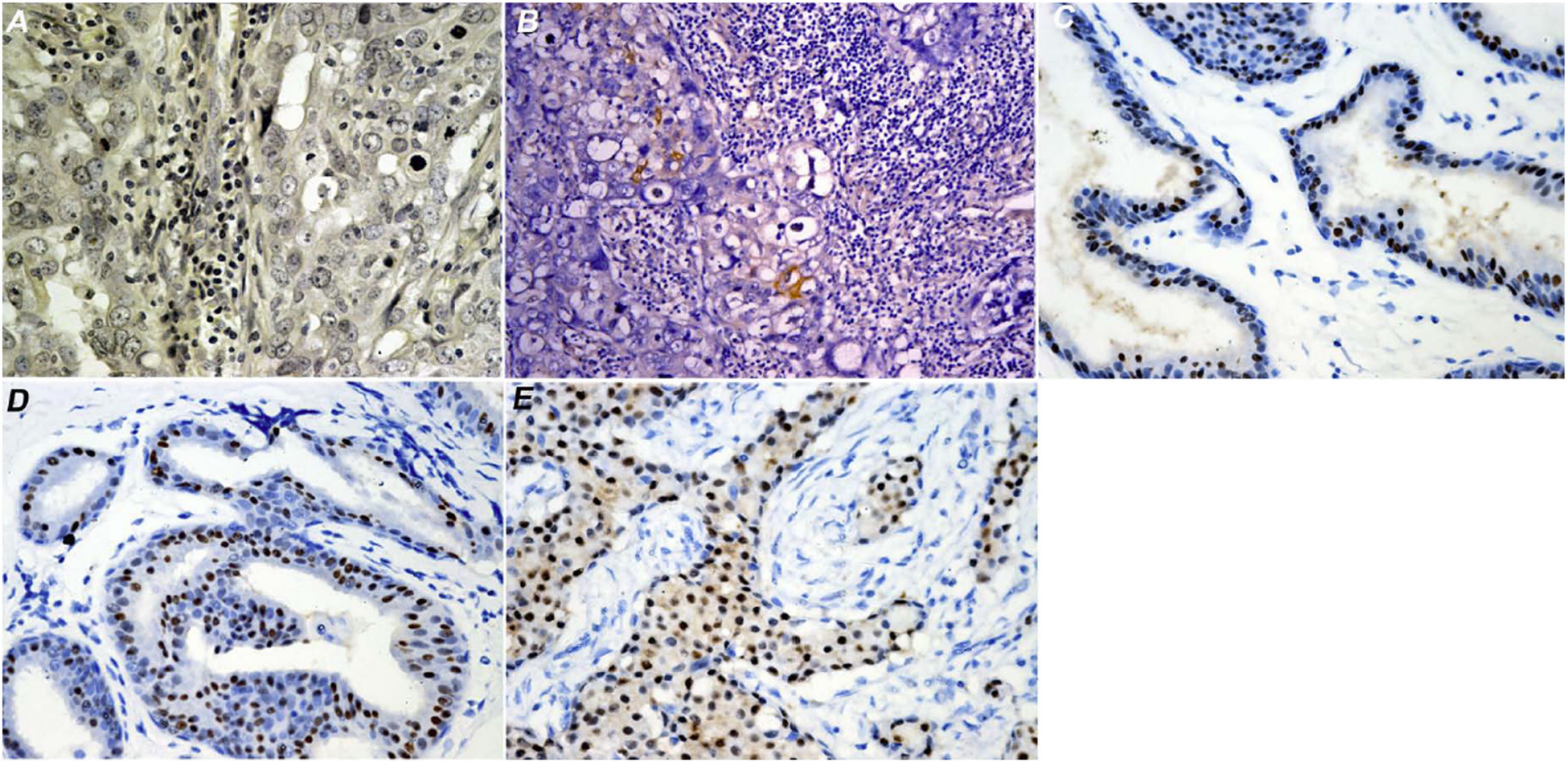
**Immunohistochemical staining of ERβ in tissues from different patients with breast cancer.** Human breast cancer section were immunostained for ERβ. **A**) All cells were negative for ERβ staining. **B**) Weak ERβ staining. **C**) Low ERβ staining (21–40% cells were positive for ERβ staining). **D**) medium ERβ staining (41–60% cells were positive for ERβ staining), and high ERβ staining (>61% cells were positive for ERβ staining).

As shown in Figure 1, no or weak staining for ERβ was presented in less than 30% of fibroadenoma tissues, whereas it was observed in nearly 40% of the breast cancer tissues. Nearly 40% of the breast cancer tissues exhibited either no staining or weak staining for ERβ compared with < 30% for fibroadenoma tissues. Negative ERβ expression was more frequently observed in breast cancer patients (18.36%) than that in the fibroadenoma group (12.42%). Among the breast cancer patients with negative ERβ expression, the proportion of patients carrying the TT genotype (32.22%), was higher than that carrying the GG (14.63%) and GT (14.72%) subgroups combined (*p* < 0.05). Similarly, among breast cancer patients with weak ERβ expression, the proportion of patients carrying the TT genotypes of rs1271572 (31.78%) was higher than for the other subgroups, GG (21.97%) and GT (20.22%) (*p* < 0.05, Figure 1). Together, these results indicated that in the breast cancer patients, the TT genotype of rs1271572 was strongly associated with negative or weak ERβ gene expression. Expression of ERβ was determined using immunohistochemical staining. Representative staining images with different scores were shown in Figure 2.

We also compared the genotype frequencies of rs1271572 (GG, GT and TT) between the ERβ–positive and ERβ–negative subgroups. The genotypes (GG, GT and TT) of rs1271572 between the ERβ–positive and ERβ–negative subgroups were found to be significantly different in cancer patients (*p* < 0.001). Although frequencies of the GG and GT carrier was not significantly different between the ERβ–positive and ERβ–negative subgroups (*p* = 0.07), the frequency of TT genotype was significantly lower (*p* < 0.001) than that of the other genotypes in ERβ-positive patients. In breast fibroadenoma patients, all genotypes and T carrier were similarly distributed between ERβ–positive and ERβ–negative breast fibroadenoma patients (*p* = 0.930 and *p* = 0.901 respectively) (Table 1).

**Table 1:** Relationship between the genotypes of rs1271572 and ERβ status in Chinese patients with breast cancer or fibroadenomas

**Cancer patients, n (%)**				**Fibroadenoma patients, n (%)**		
**rs1271572**	**ERβ–positive**	**ERβ–negative**	***p*value**	**ERβ–positive**	**ERβ–negative**	***p*value**
GG	226 (26.43)	39 (4.56)		205 (32.64)	30 (4.78)	
GT	350 (40.94)	60 (7.02)	<0.001	266 (42.36)	38 (6.05)	0.93
TT	122 (14.27)	58 (6.78)		79 (12.58)	10 (1.59)	
GG	226 (26.43)	39 (4.56)		205 (32.64)	30 (4.78)	
GT+TT(T carrier)	472 (55.20)	118 (13.80)	0.07	345 (54.94)	48 (7.64)	0.901

We next analyzed the genotypic frequencies of rs1271572 in ERβ-positive cases between patients with breast cancer and fibroadenoma. Although ERβ-positive cases were more frequently detected in fibroadenoma patients (*p* = 0.002, Table 2), ERβ-positive cases with rs1271572 TT genotype was less frequently detected in breast cancer patients (*p* < 0.001, Table 2). The genotypic frequencies of GT and GG showed no significant difference between these two subgroups.

**Table 2:** Relationship between the genotypes of rs1271572 and the ERβ–positive groups of breast cancer and fibroadenoma patients

**Groups**	**Patients, n(%)**	**GG**	**GT**	**TT**
Cancer				
ERβ–positive	698/855 (81.64)	226 /265 (85.28)	350 /410 (85.37)	122/180 (67.78)
Fibroadenoma				
ERβ–positive	550/628 (87.58)	205/235 (87.23)	266/304 (87.50)	79/89 (88.76)
OR	0.631	0.848	0.858	0.266
CI 95%	0.470–0.846	0.508–1.415	0.556–1.324	0.129–0.552
p value	0.002	0.528	0.49	<0.001

Importantly, our results strongly suggest that the TT genotype of rs1271572 may be associated with downregulation of ERβ expression in breast cancer (Figures 1 and 2, Tables 1 and 2).

### TT genotype of rs1271572 was a risk factor for breast cancer-related death

Univariate and multivariate analyses were first performed to examine whether rs1271572 genotypes were associated with clinical parameters in breast cancer. No significant association was observed between the frequency of either the homozygous TT or heterozygous GT genotypes of rs1271572 and the clinical characteristics including tumor size, menopause, age, lymph node positive, breast cancer related receptors status (ER-α, PgR, HER2) or breast cancer stage (TNM) in patients with breast cancer (Additional file 5: Table S3).

Multivariate Cox regression analyses were next performed to explore whether rs1271572 genotypes were associated with breast cancer susceptibility. As shown in Table 3, the TT genotype of rs1271572 was an independent risk factor for cancer-related death in breast cancer women (hazard ratio: 2.58; 95% CI: 1.31 ~ 3.79; *p* = 0.0065). Other statistically significant variables associated with cancer-related death included ERβ (hazard ratio: 1.67, *p* = 0.027), ER-α (hazard ratio: 0.81, *p* = 0.035), and HER2 (hazard ratio: 1.78, *p* = 0.015). Interestingly, the clinical tumor size, nodal status, TNM, menopause, age and PgR status did not show statistically significant associations with cancer-related death in this cohort of breast cancer patients.

**Table 3:** Factors influencing the risk of death using multivariate Cox regression analyses

**Variable**	**Hazard ratio**	**95% CI**	**Wald test*p*value**
Genotypes (TT *v* GT/GG)	2.58	1.31~ 3.79	0.0065
ERβ (negative *v* positive)	1.67	0.71~1.67	0.027
ERα (negative *v* positive)	0.81	0.67~0.98	0.035
HER2 (negative *v* positive)	1.78	0.76~1.87	0.015
Tumor size (<20 mm *v* ≥20 mm)	0.96	0. 62~1.51	0.951
Nodal status (positive *v* negative)	1.21	0.53~1.83	0.14
TNM (T3–4 *v* T1–2)	1.67	0.83~1.94	0.054
menopause (premenopausal *v* post)	1.26	1.03~1.78	0.058
age (≥50 *v* <50)	0.86	0.83~1.21	0.24
PgR(positive *v* negative)	0.97	0.76~1.09	0.21

Multivariate analyses revealed that the TT genotype of rs1271572, ERα and HER2, is an independent prognostic factor for risk of cancer-related death in breast cancer patients (Table 3). All these strongly suggest that TT genotype of rs1271572 is a functionally important SNP in breast cancer. Our finding that ERβ is related to a favorable outcome is consistent with previous report by of Vinayagam et al. [27].

### TT genotype of rs1271572 was associated with poor prognosis and higher risk of brain metastasis

**Figure 3:**
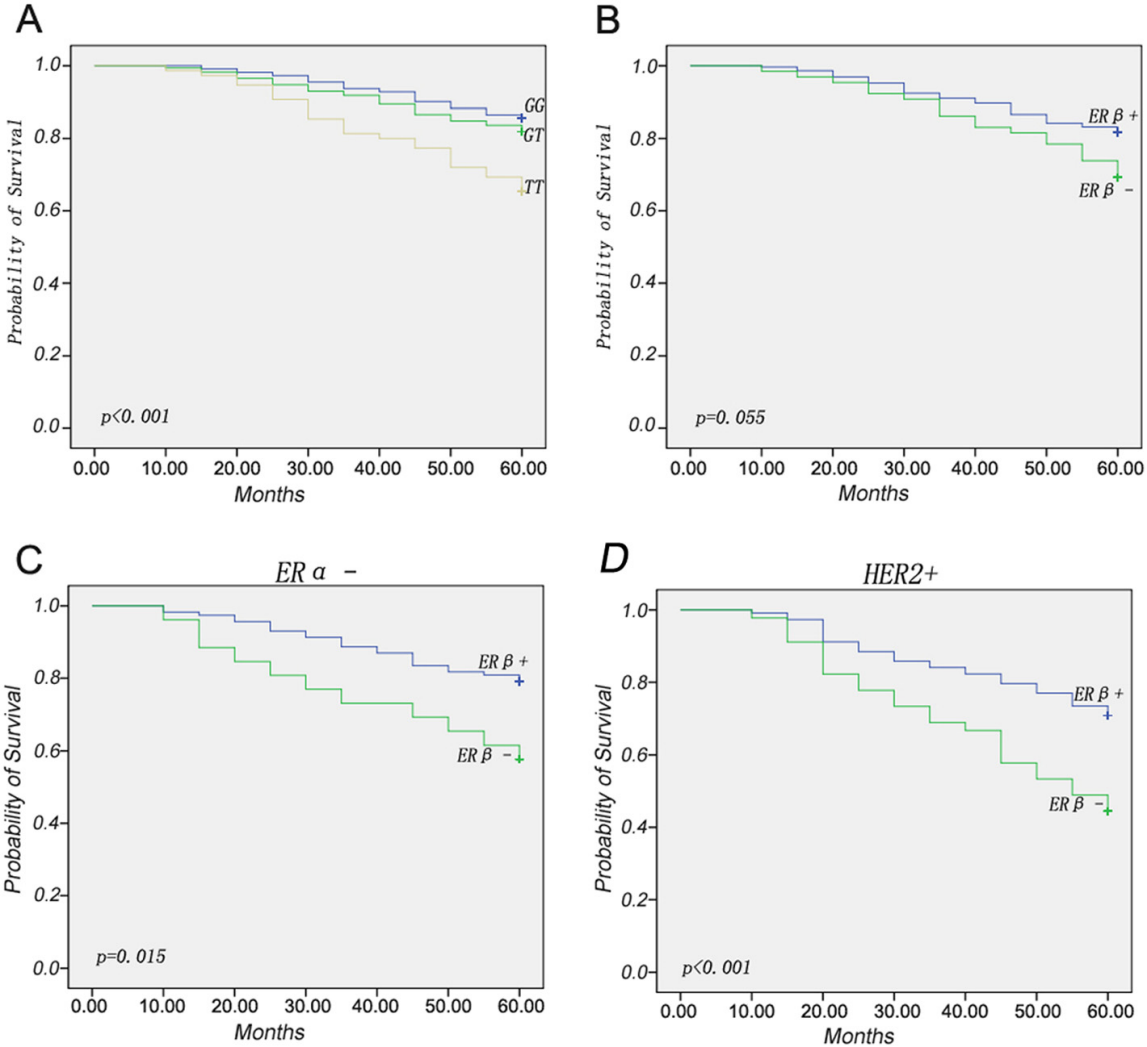
**TT genotype of rs1271572 was associated with worse survival in breast cancer.** Kaplan–Meier breast cancer-free survival was applied to determine the prognosis **A**) among patients with the three genotypes (GG, GT and TT) of rs1271572; **B**) between ERβ–positive and ERβ–negative patients; **C**) between ERβ–negative and ERβ–positive patients in the ER- subgroup, and **D**) between ERβ–negative and ERβ–positive patients in the Her + patients. The *p* values were determined using the log-rank test; a significant difference was assumed for *p* < 0.05.

The median follow up period was 43.24 (range 8.2–62.46) months for the participated cancer patients, and the mean follow up period was 49.33 months. The Kaplan-Meier survival analyses demonstrated that patients with the TT genotype of rs1271572 had a shorter survival rate than patients with the GG or GT genotypes of rs1271572 (*p* < 0.001, Figure 3A). No significant difference in survival rates between ERβ–positive and ERβ–negative patients (*p* = 0.055, Figure 3B) was observed. However, among ERα–negative and Her2-positive cases, ERβ–negative patients had a shorter survival rate than ERβ–positive patients (*p* = 0.015 and *p* < 0.001, respectively) (Figure 3C and 3D). However, among ERα–positive and Her2-negative cases, no correlations were observed between the expression levels of ERβ and survival rates (data not shown). In addition, we found that breast cancer patients with the TT genotype of rs1271572 were more likely to suffer brain metastases (29.23%) than patients with the GG (10.34%) or GT (9.47%) genotypes (p = 0.005) (Table 4). The location of distant metastases was recorded for the first metastatic organ found during the follow-up period. Data for patients with multiple organ metastasis first diagnosed during follow-up, were grouped in “other” in Table 4.

**Table 4:** The relationship of the genotypes of rs1271572 with the occurrence and location of distant metastases five years following diagnosis

	**Recurrence**	**Distant metastasis n (%)**				
	**rate n (%)**	**Lung**	**Liver**	**Brain**	**Bone**	**Other**
**Breast cancer**	218/835(26.11)	70/218(32.12)	42/218(19.27)	33/218(15.14)	52/218(23.85)	21/218(9.63)
**GG**	58/252(23.02)	20/58(34.48)	12/58(20.69)	6/58(10.34)	11/58(18.97)	9/58(15.52)
**GT**	95/404(23.51)	36/95(37.89)	17/95(17.89)	9/95(9.47)	26/95(27.37)	7/95(7.37)
**TT**	65/179(36.31)	15/65(23.08)	13/65(20.00)	19/65(29.23)	12/65(18.46)	6/65(9.23)
*p* value	0.065	0.231	0.904	0.005	0.835	0.483

Our results demonstrated that patients with the TT genotype of rs1271572 had significantly poorer five-year survival rates (Figure 3A) and were more likely to suffer brain metastases (Table 4) than patients with the other two genotypes. ERβ expression modulates adhesion and migration of breast cancer cells [28], suggesting that the TT genotype of rs1271572 might promote brain metastases of breast cancer by suppressing ERβ gene expression.

Although there was no significant difference in cancer-free survival between the ERβ–positive and ERβ–negative subgroups (p = 0.055, Figure 3B) among breast cancer patients, the TT homozygotes had poorer survival rates (p < 0.001 Figure 3A). This apparent contradiction might be because, in the TT genotype subgroup, patients with both ERβ–negative and ERβ weak expression (nearly 64%) were included. Moreover, ERβ–negative/ERα–negative breast cancer patients had significantly poorer survival rates compared with patients in the ERβ–positive/ERα–negative subgroup (p = 0.015, Figure 3C). This result is consistent with the findings of an earlier report that ERβ, particularly in ERα–negative patients, was associated with increased survival (distant disease-free survival) rates [29]. Rakha et al. recently reported that HER2+ was the poorest prognostic factor among different molecular subtypes [30]. In Her2 positive patients, ERβ–negative patients had significantly poorer survival rates compared in the ERβ–positive patients (p < 0.001, Figure 3D), suggesting that ERβ–positivity confers a better outcome for patients with the poor prognostic factors including ERα–negative and HER2 –positive. All of the results reported here support the finding that detection of ERβ status in breast cancer tissues may provide clinically useful information in addition to the well established ERα/HER2/PgR assay.

### The rs1271572 T allele may be associated with loss of Yin Yang 1 binding in ERβ promoter 0 N and suppress ERβ expression in breast cancer

**Figure 4:**
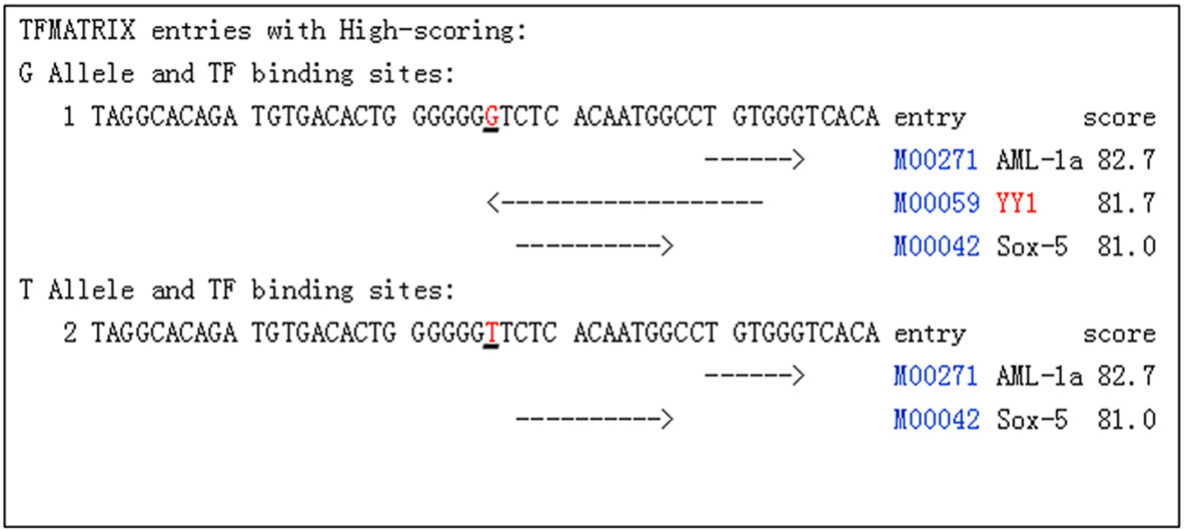
**Prediction of TF binding sites in the different alleles of rs1271572.** The results from the TFSEARCH (http://www.cbrc.jp/research/db/TFSEARCH.html) prediction of transcription factor binding sites in the ERβ promoter were shown. The T allele of rs1271572 resulted in the loss of the Yin Yang 1 (YY1) transcription factor binding site.

Transcription of the human ERβ gene occurs from at least two different promoters, promoter 0 N and promoter 0 K, with promoter 0 N being more prominent in breast epithelial cells and breast cancer cells [15][16]. As the TT genotype of rs1271572 is significantly associated with low ERβ expression in breast cancer patients (Figure 1), we hypothesized that certain transcriptional factors (TFs) that are critical to regulate ERβ expression specifically bind to G allele, but not T allele containing promoters. To search for TFs that differently bind to these promoters, bioinformatics approaches (http://www.cbrc.jp/research/db/TFSEARCH.html; http://variome.kobic.re.kr/SNPatPromoter/) were used which showed that the rs1271572 T allele could result in the loss of the Yin Yang 1 (YY1) transcription factor binding (Figure 4). We speculated that the loss of the YY1 binding site might be involved in the decreased expression of the ERβ gene.

**Figure 5:**
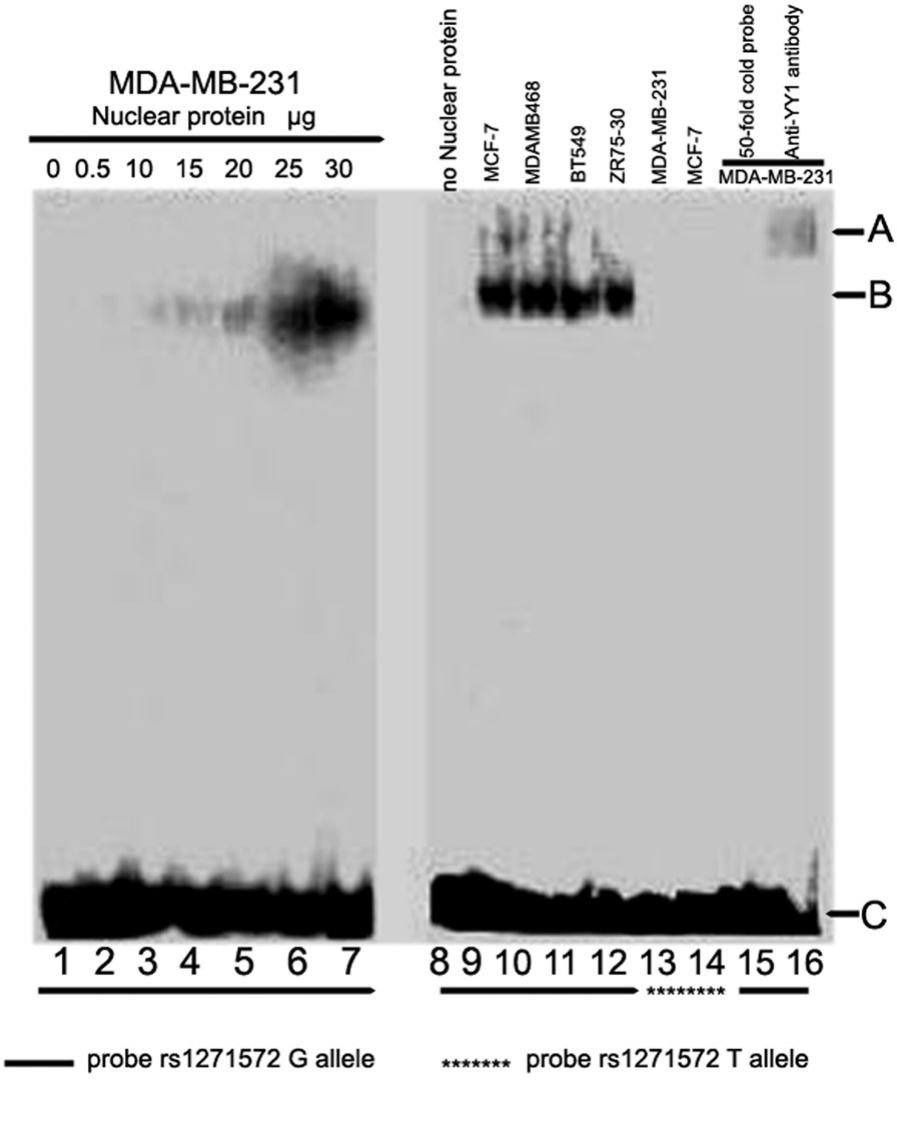
**Binding of nuclear proteins in breast cancer cells to**^**32 **^**P**- **rs1271572G DNA probes.** Different doses of nuclear proteins isolated from MDA-MB-231 cells were incubated with ^32^P-rs1271572G probes and the DNA-protein complexes were shown following autoradiography (lanes 1–7). The nuclear proteins isolated from MCF-7, MDA-MB-468, BT-549 and ZR75-30 were incubated with ^32^P -rs1271572G and the DNA-protein complexes were shown (lane 9–12). When ^32^Prs1271572T probes were used, no specific DNA-protein binding was observed (lanes 13 and 14). Binding activity of MDA-MB-231 nuclear extracts with ^32^P-rs1271572G probe was inhibited by cold rs1271572G probes(×50 times higher than ^32^P labeled probes) (lane 15). The DNA-protein complex was supershifted after further incubating with anti-YY1 antibody in MDA-MB-231 cells (lane 16). Position A indicated the binding complex in the presence of the anti-YY1 antibody. Position B indicated the binding complex of ^32^Prs1271572G DNA probes with nuclear proteins from the different breast cell lines. Position C indicated free 32P- rs1271572G DNA Probes.

To explore the role of the T allele in YY1 binding, binding activity of the two probes that contain sequence from either rs1271572G or rs1271572T was compared using EMSA in a panel of breast cancer cell lines. As shown in Figure 5, specific protein-DNA complex were detected with probe rs1271572G but not probe rs1271572T. Moreover, supershift with YY1 antibody indicated that YY1 was specifically bound to the rs1271572G oligo in these cells. Our data have shown that except for YY-1, other transcriptional factors could not bind to the region of rs1271572G and that YY-1 lost its capacity to bind to the region of rs1271572(T) (Figure 5).

**Figure 6:**
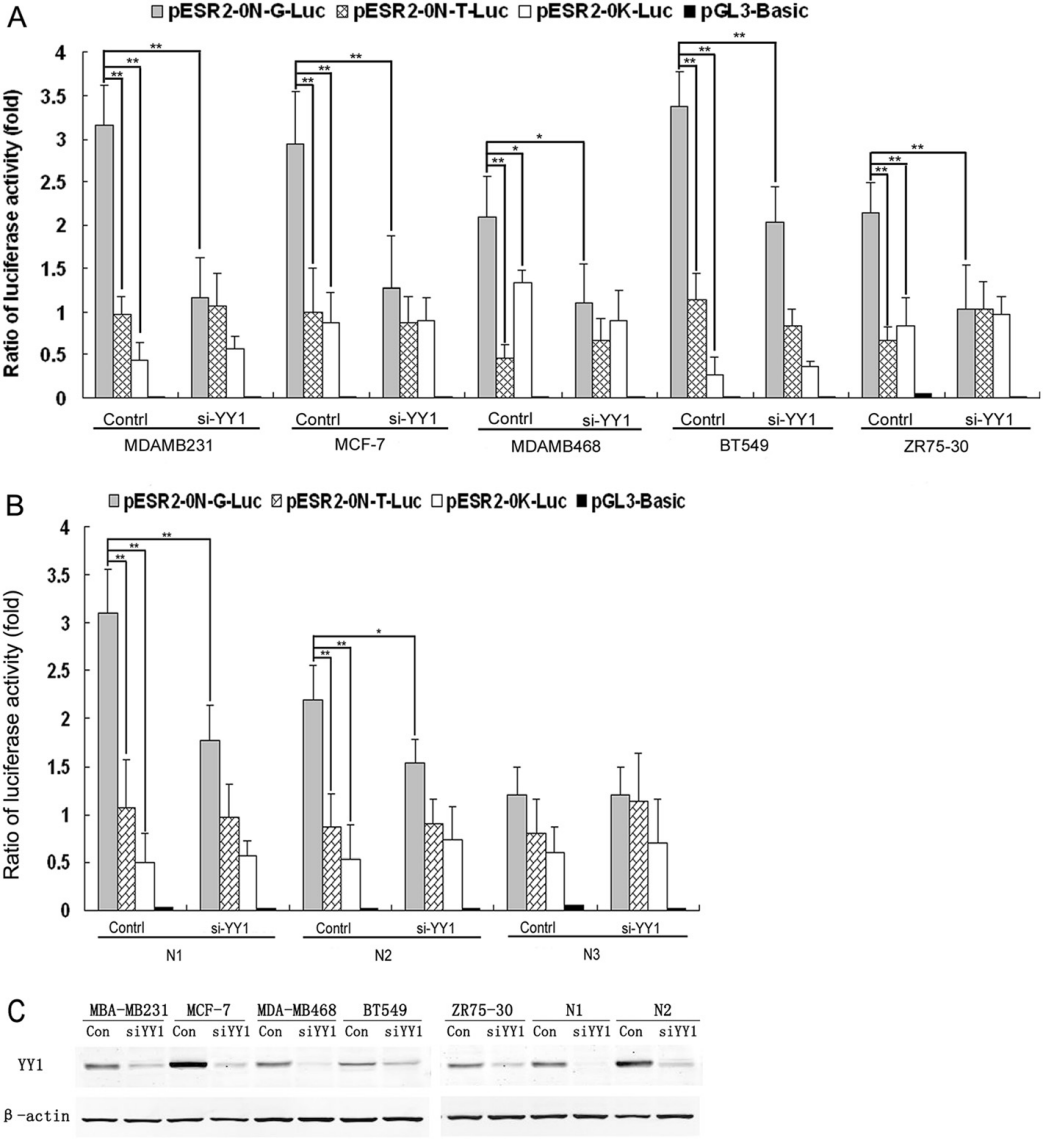
**The T allele of rs1271572 down**-**regulated the activity of the ERβ promoter 0 N.A).** The human breast cancer cells (MDA-MB-231, MCF-7, MDA-MB-468, BT-549 and ZR75-30) were transfected with the normalization control vector pRL and each of the pESR2-0 N-G-Luc, pESR2-0 N-T-Luc, pESR2-0 K-Luc (promoter 0 K) alone, or together with the YY1-specific siRNA oligos. Lucifease activities of these reporters were determined using the dual luciferase assay kit. **B**). Primary breast cancer cells (N1, N2 and N3) were transfected with the vectors as in **A**) and luciferase activity was determined. The figure represents the results of three independent experiments. *p <0.05;**p <0.01. Graphs show means + −S.E.M. (n =8). **C**). The human breast cancer cell lines (MDA-MB-231, MCF-7, MDAMB468, BT549 and ZR75-30) and the primary breast cancer cells (N1 and N2) were transfected with YY1-specific siRNA oligos and YY1 expression was examined by western blot. β-actin was used to confirm equal protein loading. Each lane was loaded with up to 30 μg of protein.

To further determine whether the T allele of rs1271572 confers decreased transcriptional activity of the ERβ gene promoter, we compared the luciferase reporter activity of pESR2-0 N-G-Luc and pESR2-0 N-T-Luc. Indeed, the reporter activity of pESR2-0 N-T-Luc was significantly lower that of pESR2-0 N-G-Luc, (Figure 6A and 6B), suggesting that the T allele of rs1271572 could lead to a decrease in the transcription activity of the ERβ gene promoter 0 N. In agreement with a previous report that transcripts from promoter 0 N were more prominent than those from promoter 0 K [16], we also observed that the luciferase activity of pESR2-0 K-Luc was significantly weaker than that of pESR2-0 N-G-Luc in the five breast cancer cell lines and in the primary cancer cells of two patients. Interestingly, luciferase activity of pESR2-0 N-G-Luc but not pESR2-0 N-T-Luc was dramatically decreased in these cells when co-transfected with YY1-specific siRNA oligos along with pESR2-0 N-G-Luc, pESR2-0 N-T-Luc or pESR2-0 K-Luc, indicating that YY1 is involved in regulation of the activity of ERβ gene promoter 0 N (Figure 6A and 6B).

Compared with GT and GG genotypes, the TT genotype of rs1271572 was associated with low ERβ expression (Figure 1). The *in vitro* luciferase assays showed that the rs1271572 G→T allele could reduce the transcription activity of promoter 0 N in ERβ gene expression (Figure 6A and 6B). These results suggest that the rs1271572 G → T allele is associated with the inhibition of expression of the ERβ gene in patients with breast cancer. Bioinformatics tools predicted that the rs1271572 G→T allele led to the loss of binding of the YY1 transcription factor (Figure 4).

Yin Yang 1 (YY1) transcription factor is highly expressed in various types of cancers and regulates tumorigenesis through multiple pathways. YY1 is generally overexpressed in breast cancer cells and tissues, and YY1 is an oncogene which negatively regulates p27. [31]. YY1 is a multifunctional protein that plays a fundamental role in normal biological processes such as embryogenesis, differentiation, replication, and cellular proliferation in vertebrates [32,33]. The reduction of inhibition of YY1 expression promoted cell migration and resulted in an invasive phenotype in breast cancer cells [34]. Pathway meta-analyses identified a number of important factors, including the YY1 transcription factor that were implicated in the metastasis of breast cancer [35]. We thus hypothesized that the TT genotype of rs1271572 suppressed ERβ expression by inhibiting YY1 binding. In support of this hypothesis, no DNA-protein complex was formed with synthetic probes that contain sequence from rs1271572T region (Figure 5). Moreover, knockdown of YY1 in breast cancer cells lines and primary breast cancer cultures also decreased the transcriptional activity of the promoter 0 N (Figure 6). Further mechanistic studies will be needed to identify additional key factors through which rs1271572 TT regulate the expression of ERβ expression in breast cancer.

## Conclusion

In this study we report that Chinese women with the TT genotype of rs1271572 had a significantly higher risk of breast cancer and have a poor prognosis and were more likely to suffer brain metastasis. The homozygous TT genotype of rs1271572 was associated with low ERβ expression in breast cancer patients. Further mechanistic studies revealed that TT genotype of rs1271572 resulted in loss of the YY1 binding site and reduced the transcription activity of the promoter 0 N in the ERβ gene.

## Abbreviations

YY1: Yin Yang 1 transcription factor; ERα: estrogen receptor alpha; ERβ: estrogen receptor beta; SNP: Single nucleotide polymorphisms; PCR: Polymerase chain reaction; EMSA: Electrophoretic mobility-shift assay; HER2: Human epidermal growth factor receptor 2; PgR: Progesterone receptor.

## Competing interests

The authors declare that there are no conflicts of interests.

## Authors’ contributions

LC designed and performed research experiments, analyzed data, and wrote the manuscript, YL collected patients data and samples, performed experiments, JQ, LZ and XC performed some experiments, analyzed the data, provided cells and reagents, XL and JJ reviewed and edited the manuscript, provided suggestions to the research. All authors read and approved the final manuscript.

## Supplementary Material

Additional file 1: Figure S1 Structure of the human ERβ gene. Exons are represented by boxes and introns by lines. The number below each box indicates the size of the exon (bp); the number above each line indicates the size of the intron (bp). Click here for file

Additional file 2: Table S1 PCR primers used for the ERβ gene SNP analyses. Click here for file

Additional file 3: Figure S2 Schematic representation of the 5’ untranslated region of human ERβ. The translation start site is indicated by ATG. A of the first codon ATG is assigned nucleotide number +1. Exons are represented by gray boxes and introns by lines. The dotted lines between the gene promoter and the DNA fragment indicate how the fragments were constructed. ESR2-0 N+indicates the DNA sequence of the ERβ promoter 0 N and the inserted partial upstream sequence of the intron to ATG of exon 1. ESR2-0 K+indicates the DNA sequence of the ERβ promoter 0 K and the inserted partial upstream sequence of intron to ATG of exon 1. Click here for file

Additional file 4: Table S2 Genotype frequencies for SNPs in the ERβ gene promoter in Chinese breast cancer patients, fibroadenoma patients and blood donors. Click here for file

Additional file 5: Table S3 Relationship between the genotypes of rs1271572 and clinicopathological factors in patients with breast cancer. Click here for file
