# Supplementary figures and images for: Significance of rs1271572 in the estrogen receptor beta gene promoter and its correlation with breast cancer in a southwestern Chinese population
Source: J Biomed Sci. 2013 May 28;20(1):32. doi: 10.1186/1423-0127-20-32 (PMC3672062; doi:10.1186/1423-0127-20-32)

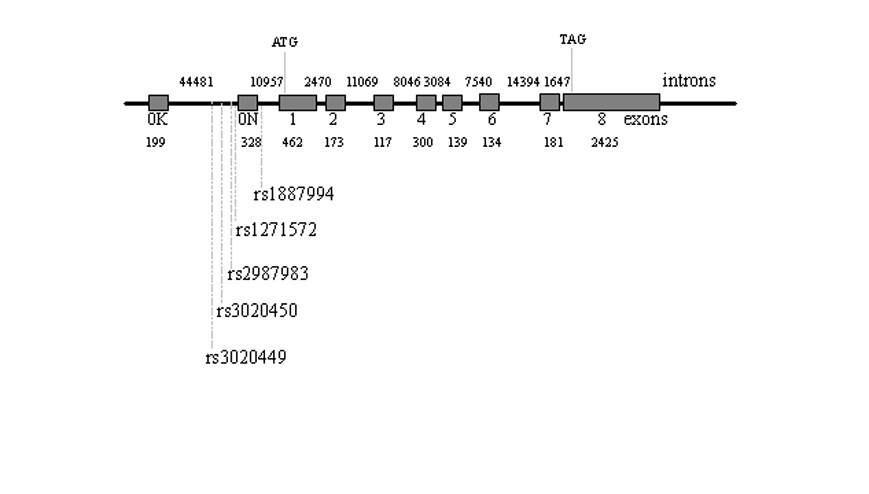

Supplement: Additional file 1: Figure S1 — Structure of the human ERβ gene. Exons are represented by boxes and introns by lines. The number below each box indicates the size of the exon (bp); the number above each line indicates the size of the intron (bp). [file 1423-0127-20-32-S1.tiff]

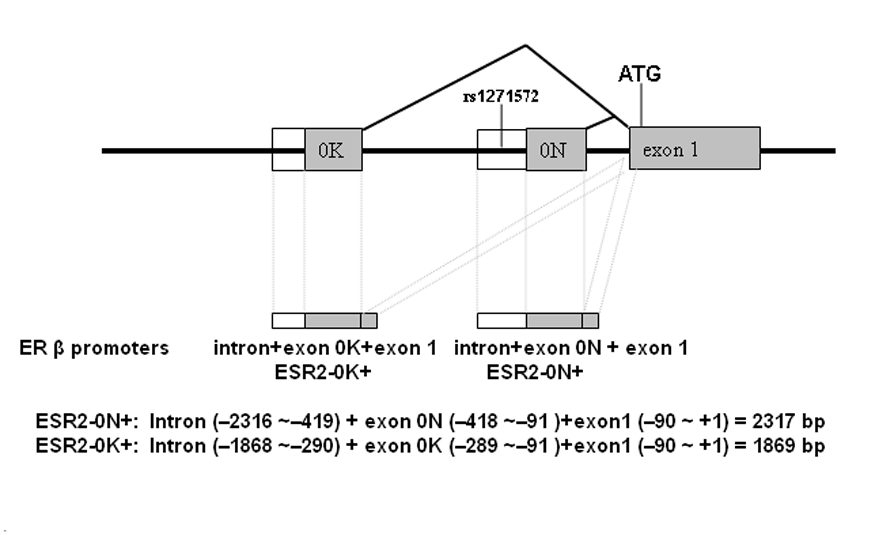

Supplement: Additional file 3: Figure S2 — Schematic representation of the 5’ untranslated region of human ERβ. The translation start site is indicated by ATG. A of the first codon ATG is assigned nucleotide number +1. Exons are represented by gray boxes and introns by lines. The dotted lines between the gene promoter and the DNA fragment indicate how the fragments were constructed. ESR2-0 N+indicates the DNA sequence of the ERβ promoter 0 N and the inserted partial upstream sequence of the intron to ATG of exon 1. ESR2-0 K+indicates the DNA sequence of the ERβ promoter 0 K and the inserted partial upstream sequence of intron to ATG of exon 1. [file 1423-0127-20-32-S3.tiff]
